# Method‐Specific Attributes that Influence Choice of Future Contraception Among Married Women in Nairobi's Informal Settlements

**DOI:** 10.1111/sifp.12070

**Published:** 2018-08-20

**Authors:** Joyce N. Mumah, John B. Casterline, Kazuyo Machiyama, Marylene Wamukoya, Caroline W. Kabiru, John Cleland

## Abstract

Despite an extensive evidence base on contraceptive method choice, it remains uncertain which factors are most influential in predisposing women toward certain methods and against others. This paper addresses this gap in knowledge by making use of rarely‐measured perceptions about specific methods, perceived social network experience of methods, and women's own past experiences using specific methods. We draw on baseline data from the project, “*Improving Measurement of Unintended Pregnancy and Unmet Need for Family Planning.”* Using conditional logit analysis, we ascertain which perceived method‐specific attributes, including past experience of methods by women themselves and by their friends, predict preferred future contraceptive method among 317 women living in Nairobi slums who are using no method but intend to start in the next 12 months. Results show that satisfaction with past use, positive experience of use by a woman's social network, husband/partner's approval, lack of interference with menses, and perception of safety for long term use were all associated with choice of a future method.

## INTRODUCTION

Over the past half century, a huge literature on contraceptive method choice has been generated including several edited volumes (Russell et al. 2000; Bulatao et al. 1989; Sundari Ravindran et al. 1997) and reviews (Daniele et al. 2017; Williamson et al. 2009; Wyatt et al. 2014). The major strands of evidence on factors that influence method choice fall into the following categories: information, availability, access, and affordability (Tsui and Ochoa 1992; Korachais et al. 2016); counseling, client provider interactions, and provider bias, including studies in Kenya (Kim et al. 1998); sociodemographic correlates of method‐choice, again including studies in Kenya and neighboring Tanzania (Magadi and Curtis 2003; Chen and Guilkey 2003); desired attributes of methods and acceptability (Snow et al. 1997; Keller 1979); the behavior and views of sexual partners and members of persons’ social network (Godley 2001; Kimuna and Adamchak 2001); and a very large number of publications on opinions and fears about specific methods among Kenyan women and, less commonly, among men (Kamau et al. 1996).

Despite this extensive evidence base, it remains uncertain which factors are most influential in predisposing women toward certain methods and against others. In this paper, we start to address this gap in knowledge by measuring perceptions about specific methods, experience with particular methods by friends and neighbors, and women's own past use of methods. Drawing on a prior literature search, we include measurement of specific perceptions concerning the possible effect of methods on menstrual disruption and fertility impairment, and their safety for long term use, in addition to unspecified health damage and side effects. We further assess the relative strength of association of these measures with the method preferred for future use among women who are currently not contracepting but intend to do so in the next 12 months. To the best of our knowledge, this is the most detailed quantitative investigation to date conducted in a low‐ or middle‐income country of factors that incline women to choose specific methods for future use and is a valuable complement to the qualitative literature.

The study draws on baseline data collected in urban slums in Nairobi, Kenya as part of the multi‐country prospective study, *Improving Measurement of Unintended Pregnancy and Unmet Need for Family Planning*, which was conducted in Nairobi, rural Kenya, and Bangladesh. The rationale and protocol for this study have been published elsewhere (Machiyama et al. 2017), but in summary it seeks to advance knowledge of unmet need and unintended pregnancy, including contraceptive uptake and continuation. Causal factors hypothesized to influence these outcomes are measured at baseline and their relative power to explain subsequent reproductive and contraceptive behavior will be assessed by follow‐up surveys. An innovative feature of the study is its detailed measurement of method‐specific perceptions that may influence decisions to adopt or continue to use specific methods and, indeed, may deter women from using any method. Drawing on this rich measurement, this paper addresses the question: which perceived method‐specific attributes, including past experience of methods by women themselves and by their friends, determine the preferred contraceptive method for a future use among women living in Nairobi slums who are using no method but intend to start in the next 12 months?

## METHODS

### Study Setting

This study focuses on two slums in Nairobi—Viwandani and Korogocho—where the African Population and Health Research Center (APHRC) maintains the Nairobi Urban Health and Demographic Surveillance System (NUHDSS). The NUHDSS covers 14 villages in both slum settlements with a population of about 65,000 individuals living in about 24,000 households in the two settlements (Beguy et al. 2015). Both settlements are characterized by high levels of unemployment, sub‐standard and overcrowded housing, limited education and social services, high levels of crime and insecurity, and inadequate water and sanitation infrastructure.

Households covered by the NUHDSS are visited twice a year to collect data on key sociodemographic and health measures including births, deaths, migration, immunization, livelihoods, as well as household amenities and assets. The NUHDSS thus provides a platform for more focused nested studies investigating the inter‐linkages between poverty and health and other outcomes.

#### Data

This study utilizes baseline data from a two‐year prospective study among a cohort of randomly selected married or cohabiting women between the ages of 15–39 years who were living in the demographic surveillance area. The upper age limit and enrollment of women in stable unions was based on the need to recruit women who were at potentially high risk of pregnancy during the observation period. Baseline data were collected from September to October 2016. The desired sample size of 2,600 was based on the formula developed by Fleiss et al. (2013), for detecting a 30 percent difference between current users and non‐users at 95% confidence level and 80 percent power with an assumption of 10 percent non‐response rate and 45 percent attrition over two years. A random sample of 5,905 women was generated from the 2016 NUHDSS database. Of these, 2,812 women were successfully interviewed during the first round of data collection, 1,917 had out‐migrated or were temporarily away, 592 were ineligible, and 584 were either not found after three attempts, their whereabouts were unknown, or they had died.

Face‐to‐face interviews with eligible women were carried out in Swahili by trained fieldworkers using electronic data capture. The survey instrument included questions on women's sociodemographic characteristics; reproductive history; sexual activity; retrospective and prospective measures of fertility preferences; perceived partner's fertility preferences; perceived risk of pregnancy; generic attitudes towards pregnancy‐prevention; method‐specific attitudes and experiences of contraceptives; and contraceptive use.

The study is particularly innovative in its extensive measurement of perceptions and attitudes toward specific contraceptive methods and this feature provides the focus of the paper. A battery of 12 questions elicited perceptions about each of eight main methods (pills, injectables, implants, intrauterine device (IUD), condoms, female sterilization, periodic abstinence, and withdrawal). These questions were asked of all women who had heard of the particular method, including current, past, and never‐users.

Ethical approval to conduct this study was granted by the AMREF Ethics and Scientific Review Committee, and the Population Council's and London School of Hygiene and Tropical Medicine's Institutional Review Boards. All women interviewed in the study gave written informed consent to participate in the study.

#### Analysis

The aim of this study was to ascertain which perceived method‐specific attributes predict future intentions to use one of the three methods that are commonly used in this setting (oral contraceptives or pills, injectables, and implants) by women who were not currently using a method but intended to do so in the next 12 months and were aware of all three contraceptive methods—oral contraceptives or pills, injectables, and implants) (n=317). These attributes include familiarity, access, perceived effectiveness, safety, side effects, ease of use, satisfaction with use among social networks, husband/partner approval, interference with menstruation, infertility, and past use. As a result of the selectivity of the sample due to our selection criterion, we assessed the sample to ascertain whether women retained in our analysis (non‐users) were statistically different from those excluded (current users). Results indicate that the characteristics of non‐users were not very different from current users (table not shown).

Following descriptive analysis, we conducted regression analysis to establish which variables were significantly associated with future method choice, net of the effects of other variables. In addition to the generic and method‐specific attributes, the data offer a large set of plausible explanatory variables including sociodemographic characteristics and fertility preferences. From the standpoint of regression modeling, the explanatory variables fall into two general classes:

Respondent [*i*] characteristics [***X_i_***]: Number of living children, schooling, fertility preferences, etc.

Attributes of each contraceptive method [*m*], specific to each respondent [*i*] [***Z_im_***]: perceived efficacy, health side effects, husband/partner's approval, and so on.

From a conceptual standpoint, the effects of respondent characteristics ***X_i_*** can be specified as effects on method versus method contrasts; for example, one can consider the effect of schooling on the choice of implant versus injectable. The effects of method attributes ***Z_im_*** are naturally specified as generic effects on method choice; for example, one can consider whether, net of other factors, women are less likely to choose methods that are thought to cause unpleasant side effects.

More formally, we modelled the probability of choice of method *m*

$$\mathrm{Pr}\left(m\right)=\beta_{m}X+\gamma Z_{m}$$

where

*m*: method *m*
*X*: respondent characteristics
*Z*: method attributes, specific to method *m*
*β*: array of effects of *X* on method *m* versus *j*
*γ*: effect of attribute *Z*

This mix of explanatory variables—respondent characteristics and perceived method attributes—is awkward to accommodate in conventional multinomial logit modeling. We therefore used the “conditional logit” model developed by McFadden (McFadden 1974; Greene 2017), which is a well‐established tool for analyzing discrete choice, such as one among a set of contraceptive methods, and indeed has previously been applied in research on contraceptive choice (Delavande 2008; Agyei‐Baffour et al. 2015). The model can be expressed as:

$$\mathrm{Pr}\left(y_{i}=m\right)=\frac{\exp(Z_{im}\gamma +X_{i}\beta_{m})}{\sum_{j=1}^{J}\exp(Z_{ij}\gamma +X_{i}\beta_{j})}$$

where notation is as above. Note that there is only one coefficient *γ* for the effect of each contraceptive attribute *m*, and a set of coefficients *β_m_* for the effects of each respondent characteristics *X* on method *m*. The latter are coefficients for each method contrast, with one method selected as reference category. We used the injectable as reference category, so there are a set of coefficients *β_m_* for the effects of *X* on the likelihood of choosing the implant rather than injectable and another set of coefficients *β_m_* for the effects of *X* on the likelihood of choosing the pill rather than injectable. These coefficients *β_m_* are as obtained in conventional multinomial logit modeling, it is coefficient *γ* that is the special contribution of the McFadden conditional logit model. Estimation is via maximum likelihood as implemented in the Stata *clogit* procedure.

In the conditional logit modeling, we considered which method attributes were significantly associated with future choice of a contraceptive method among current non‐users who intended to use either pills, injectables, or implants in the next 12 months. Concurrently, we also estimated the effects of respondent stage of reproductive career (age, number of living children), socioeconomic status (educational attainment), and current fertility preferences.

## RESULTS

### Descriptive Results

Table 1 shows selected background characteristics of the women interviewed during the baseline by the contraceptive use status. Seventy‐seven percent of the women in our sample were aged 25–39 years, and 23 percent were between the ages of 15 and 24 years. About 33 percent of non‐users reported a completed primary education, while 27 percent reported no formal or incomplete primary education.

**Table 1:** Percent distribution background characteristics of married or cohabiting women aged 15–39 years living in two urban slums in Nairobi, 2016

	Current User		Current Non‐User		Total	
	%	n	%	n	%	n
**Current age**						
15–24	23.3	493	22.5	156	23.1	649
25–39	76.7	1,625	77.5	538	76.9	2,163
**Highest level of education**						
No education/some primary	19.0	402	27.4	190	21.1	592
Completed primary	41.4	876	33.3	231	39.4	1,107
Secondary+	39.7	840	39.3	273	39.6	1,113
**Ethnicity**						
Kikuyu	25.2	534	21.3	148	24.3	682
Luhya	17.8	378	16.3	113	17.5	491
Luo	15.1	319	16.0	111	15.3	430
Kamba	25.0	530	20.3	141	23.9	671
Other	16.9	357	26.1	181	19.1	538
**Fertility Preference**						
Want soon/want within 2 years/undecided	15.7	333	42.4	294	22.3	627
Want to wait 2+ years	51.4	1,088	34.9	242	47.3	1,330
Wants none/no more	30.1	638	20.3	141	27.7	779
Other preference^	2.8	59	2.4	17	2.7	76
**Current contraceptive use**						
Injectables	43.0	910	–	–	32.4	910
Implants	26.3	557	–	–	19.8	557
Pills	11.1	235	–	–	8.4	235
Other methods	19.6	416	–	–	14.8	416
Not using	0.0	0	–	–	24.7	694
Total	100.0	2,118	100.0	694	100.0	2,812

NOTE: ^Other fertility preferences include sterilized, cannot get pregnant, other preference, not asked.

About 75 percent of the women were currently using a contraceptive method at the time of the baseline survey. Among current users, injectables were the dominant method currently being used (43 percent) followed by implants (26 percent) and pills (11 percent). About 25 percent of the women in the sample were not currently using any method at the time of the baseline interview.

About 42 percent of non‐users wanted another child very soon, within two years or were undecided about their preference, about 35 percent wanted to wait for two or more years, while 20 percent wanted no children or no more children. In contrast, about half of current users wanted to wait for two or more years before having another child, about 30 percent wanted no children or no more children and 16 percent wanted another child very soon, within two years or were undecided about their preference.

Women who were not currently using a method were asked if they intended to use a method in the next 12 months, or in the more distant future, and which method they would prefer to use. Table 2 provides the percentage distribution of methods non‐users would prefer to use in the future among those intending to use a method in the next 12 months. Overall, about 60 percent of non‐users reported that they intended to use a method within 12 months. Among these 416 women who intended to use a method in the next 12 months, half chose injectables, 19 percent chose implants and 10 percent chose pills.

**Table 2:** Percent distribution of methods non‐users would prefer to use in the future among those intending use in the next 12 months—Nairobi, 2016

	Total	
	%	n
**Intention to use among non‐users** a		
Intend to use in <12 months	59.9	416
Intend to use in 12+ months/Do not intend to use	39.8	276
**Total**	100.0	694
**Method intend to use among those who intend to use in <12 months**		
Injectables	50.5	210
Implants	18.8	78
Pills	9.6	40
Other methods	21.2	88
**Total**	100.0	416

NOTE: ^a^2 women were not asked about intention to use.

#### Women's Perceptions About the Three Most Preferred Methods

Table 3 summarizes views on and past experience with injectables, pills, and implants among non‐users who intended to use one of these three methods in the next 12 months. Results show that very few women thought any of the three methods were difficult to obtain. With regards to effectiveness of the method, about 36 percent of women thought pills were not effective at preventing pregnancy compared to 12 percent who said the same for implants and 11 percent for injectables. Concerns about unspecified serious health problems were more common for implants and injectables than for pills, and almost a third of women reported that these two methods might cause infertility. Most (78 percent and 62 percent, respectively) women thought (correctly) that injectables and implants interfered with menstruation. Similarly, close to half believed that each of the three methods cause unpleasant unspecified side effects, and a majority considered it unsafe to use each of the three methods for a long time without a break.

**Table 3:** Percentage of respondents with specific negative opinions about selected methods, among married women aged 15–39 years who were not currently using a method and intended to use injectables, implants, or pills within the next 12 months in Nairobi, 2016

	Injectables		Implants		Pills	
Negative opinions	%	n	%	n	%	n
**Hard to obtain**	2.2	7	8.5	27	7.3	23
**FP use among social network**						
None	3.5	11	6.3	20	19.6	62
Don't Know	1.9	6	1.3	4	1.3	4
**Experiences of friend, relatives or neighbors**^						
Unsatisfactory	11.0	33	18.4	54	33.5	84
**Not effective at preventing pregnancy**	11.0	35	12.3	39	36.3	115
**Cause serious health problems**	17.4	55	19.6	62	12.0	38
**Interfere with menstruation**	76.7	243	61.8	196	41.3	131
**Cause unpleasant side effects**	56.5	179	57.1	181	44.2	140
**Unsafe to use for a long time (should take a break)**	60.3	191	52.7	167	68.5	217
**Causes infertility**	29.7	94	25.9	82	15.8	50
**Husband disapproves**	19.6	62	38.5	122	39.7	126
**Past use**						
Never user	23.3	74	82.3	261	63.4	201
Past user‐satisfied	49.2	156	11.4	36	16.7	53
Past user‐not satisfied	27.4	87	6.3	20	19.9	63
**Mean number of negatives**	3.49	–	3.48	–	4.06	–
**TOTAL (N)**	**100.0**	**317**	**100.0**	**317**	**100.0**	**317**

NOTES: Of the 416 women who were not currently using a method and intended to use any method in the next 12 months, there are 317 women who intended to use either injectables, implants, or pills, who knew all three methods, and who stated that they could get pregnant.

^This panel only includes the women for whom most/about half/few members of social network had used the method.

Women were also asked if their friends, relatives, and neighbors (social network) had used the three methods and whether or not their experience had been satisfactory. A high proportion of women reported their network had used injectables (69 percent), followed by implants (39 percent) and pills (20 percent). Though the level of dissatisfaction was overall low for these methods, it did vary by the three methods: for most women, dissatisfaction within their social network was highest for users of pills (34 percent), followed by implants (18 percent) and injectables (11 percent).

We also asked women whether their husband/partner would approve or disapprove of using any of the three methods if they wanted to avoid a pregnancy. Even though only 13 percent of husbands were perceived to disapprove of family planning in general (result not shown), about 40 percent of women thought that their partner would disapprove of implant or pill use while 20 percent would disapprove of injectable use.

Among women who intended to use any of the three methods in the future, 23 percent of women were never‐users of injectables, compared with 82 percent of women who had never used implants and 63 percent who had never used pills. Most past users of injectables and implants, but less than half of past pill users reported satisfaction with the method. In the whole sample of non‐users, 27 percent were dissatisfied past users of injectables and 20 percent were dissatisfied past pill users.

### Conditional Logit Analysis of Future Method Choice

Table 4 presents the results from the conditional logit regression analysis based on data from women who were not currently using contraception and expressed an intention to use either injectable, implant, or pills in the next twelve months. The table shows the effects of both method attributes (top panel) and effects of respondent characteristics (lower panels). This discussion focuses on the former, which are the distinctive contribution of this research. Table 4 contains two models, one that excludes husband/partner's approval (Model 1) and a second that includes husband/partner's approval (Model 2). Comparison of the two sets of estimates tests the robustness of the results to exclusion or inclusion of this variable that one might posit serves as a proxy for the respondent's overall receptivity to the method (due to whatever considerations that may not have been tested or captured by the tool or analysis). This would be a type of “halo effect” (i.e. reported perceptions of attributes for which evidence is lacking or ambiguous are driven by positive or negative evaluations of other attributes; (see (Van Doorn 2008)).

**Table 4:** Intention to use injectables, implants, and pills within 12 months among non‐users and pregnant women who know all three methods—Nairobi 2016

	**Model 1 Excludes husband/partner's approval**	**Model 2 Includes husband/partner's approval**
	**OR**	**OR**
n	**317**	**317**
**Effects of Method Attributes**		
Easy to find	1.75	1.50
Social network tried and satisfied	1.92^a^	1.80^a^
Effectively prevents pregnancy	2.11^a^	1.88±
Doesn't cause health problems	1.18±	1.11
Doesn't interfere with menses	2.05^a^	2.15^a^
Doesn't cause side effects	0.92	0.86
Safe for long use	1.73^a^	1.63±
Doesn't cause infertility	1.73±	1.50
Past use and satisfaction (Ref: Never used)		
Ever used + satisfied	3.56^a^	2.97^a^
Ever used + dissatisfied	1.02	0.84
Husband approves of method		3.20^a^
**Injectable (reference category)**	* *	* *
**Effects on Choice of Implant (vs. Injectable)**		
Slum of residence (Ref: Korogocho)	0.65	0.64
Age group (Ref: 15–24 years)	0.36^a^	0.37^a^
Education attainment (Ref: None/Incomplete primary)	1.04	1.08
Number of living children	0.94	0.94
Fertility preference (Ref: Wants child: soon/within 2 years/undecided)		
Wants child: wait 2+ years	0.87	0.91
Doesn't want a/another child	2.82^a^	2.88^a^
**Effects on Choice of Pill (vs. Injectable)**		
Slum of residence (Ref: Korogocho)	1.12	1.11
Age group (Ref: 15–24 years)	0.92	1.00
Education attainment (Ref: None/Incomplete primary)	1.39	1.42
Number of living children	1.34±	1.31±
Fertility preference (Ref: Wants child: soon/within 2 years/undecided)		
Wants child: wait 2+ years	1.10	1.10
Doesn't want a/another child	0.87	0.93

a Significant at p < 0.05; ^**^p < 0.01; ^***^p < 0.001; ±p< 0.10

In the model that excludes husband/partner's approval, results show that if members of the woman's social network tried and was satisfied with any of the methods, the odds of choosing that method increased by 1.9 times compared with women whose social network tried but had dissatisfied experience with that method. Similarly, if the women believed that the method effectively prevented pregnancy, her odds of choosing the method increased twofold. Further, if a woman believed a method does not interfere with menses, she had twice the odds of choosing the method compared to the woman who believed the method interferes with menses. Methods that were perceived to be safe to use for a long time without taking a break were associated with 1.7 times higher odds, while women who had ever used a method and were satisfied had 3.5 times higher odds of choosing the method compared with women who had never used the method. Though marginally significant, women who perceived that a method did not impair fertility were 73 percent more likely to choose it than those who believed that the method might cause long term infertility.

Neither unspecified health problems nor side effects were significantly associated with method preference. Because menstrual disruption, concerns about infertility, and perceived safety for prolonged use are components of overall health problems and side effects, we re‐ran the model without these three factors. However, the effects of unspecified health concerns and side effects remained insignificant, with p‐values of 0.23 and 0.26, respectively (results not shown).

In the second model, husband/partner's approval was included as one of the attributes. The inclusion of this variable has some bearing on the statistical significance of estimated effects of several method attributes. Specifically, the p‐values for the effects of perceptions that a method does not effectively prevent pregnancy, does not cause unspecified health problems, and is safe to use for a long time are all reduced when the husband/partner's approval is included, suggesting that some of the impact of these method attributes reflects the woman's husband/partner's approval. But these are relatively small changes in the estimates. Overall, addition of husband/partner's approval to the regression equation hardly changes the estimated effects of other method attributes. Note that if a woman believed that her husband approves of the method (as compared to disapproves), she was over three times more likely to choose the method. Husband/partner's approval, as reported by the respondents, is a powerful predictor of method choice. Because the effects of other variables in the regression hardly change upon introduction of husband/partner's approval, the results suggest that the influence on method choice of perceived husband/partner's approval is genuine rather than a proxy for other concerns and considerations.

In both models 1 and 2, women between the ages of 15 and 24 years had 74 percent lower odds of choosing implants compared to injectables. Similarly, women who did not want a child or any more children had 2.8 times higher odds of choosing implants compared with injectables.

## DISCUSSION

Free and informed choice of contraceptive method is one of the fundamental principles of family planning provision. While it is well established that counseling can make a difference, most recently from the multi‐country CHOICE study (Bitzer et al. 2012), the impact on choice of women's opinions and perceptions about methods, as well as her past experience with use of methods, is poorly understood. The central aim of this paper is to advance our knowledge of the relative influence of these perceptions and past experience on intentions to use specific methods in the future.

In this slum population, as in most other populations, the current method mix is skewed. Three methods—injectables, implants and pills—comprised 80 percent of all use. One‐quarter of women were using no method for a variety of reasons that we have not described but include desire for another child, current pregnancy, post‐partum amenorrhea as well as unmet need. Of these non‐users, 60 percent reported an intention to adopt a method in the next 12 months. The profile of intentions closely mimics current method‐mix: 79 percent specified injectables, implants, or pills as their preferred method and the rank order of preference among these three is exactly the same as their relative popularity among current users. This degree of skewness justifies our focus on these methods. While it would have been of great interest to examine minority preferences, for instance for IUDs or sterilization, there were simply too few relevant cases to sustain quantitative analysis.

In this population, non‐users who intended future use recorded considerable prior contraceptive experience. Three‐quarters had used injectables, 37 percent pills, and 18 percent implants. Most knew at least some friends, neighbors, or relatives who had used each method. The perceptions about methods are thus based partly on personal experience and partly on information and judgments derived from social networks or other sources.

In a high contraceptive prevalence setting, we anticipated that the views of the three main methods would be favorable, at least among women intending to use any one of them. However, it is clear that high levels of current and past use do not necessarily imply positive perceptions in the population. Most women in our study sample believed that prolonged use of each method without taking a break was unsafe and acknowledged that injectable and implant use would interfere with regular menses. Further, close to half thought that each method caused unspecified unpleasant side effects; and appreciable minorities considered that infertility or unspecified serious health problems could be a consequence of use. The overall impression is that modern contraceptive use is regarded by many with anxiety about its effect on health and in anticipation of side effects. Results corroborate findings from a recent study which showed contraceptive discontinuation is high among this population (Mumah et al. 2015).

The women's perceptions of their husband/partner's method‐specific approval emerged as a powerful net predictor of future method choice. This is reflective of the fact that while only 13 percent of women thought that their partner disapproved of contraception in general, close to 40 percent reported spousal disapproval of pills and implants and 20 percent disapproval of injectables. In advance, we worried that stated method‐specific partner approval proxied for other method‐specific attributes, and hence regressions were estimated that excluded and included husband/partner's approval. In the event, the effects of other variables hardly differed between models excluding and including the husband/partner's approval, an outcome that on the face of it suggests that the effects of this variable are genuine. While the possibility that husband/partner's approval proxies for other unmeasured contraceptive attributes cannot be ruled out, the set of attributes measured in the Nairobi data is relatively comprehensive.

Other than husband/partner's approval, the single largest influence on future method choice was satisfaction with past use. This result comes as no surprise but underscores the importance of having readily available alternatives for women who have tried a method but disliked it. Though most past injectable and implant users reported a satisfactory experience, over one‐quarter of the entire sample of non‐users had found injectable use experience to be unsatisfactory. Past users of oral contraceptives (pills) had a less positive experience than past users of the other two methods; 20 percent of the sample were dissatisfied past users of this method.

Never‐users of a method were inevitably more reliant on the opinions of their friends, relatives and neighbors. Nearly all respondents knew individuals in their social network who had used each method and two‐thirds perceived their experience of injectables and implants as positive, though this proportion drops to about half for pills. The perception of satisfactory experience with a method within a woman's social network was a strong influence on her own method preference. This result is consistent with a large body of both statistical and qualitative evidence of the importance of social influences on contraceptive decisions (Rutenberg and Watkins 1997; Montgomery and Casterline 1993; Montgomery and Chung 1999; Entwisle et al. 1996). The power of social influence goes a long way towards explaining why the range of methods used in most populations is so narrow. Most women are not contraceptive innovators; on the contrary, most prefer familiar methods that their friends have used with success.

Generalized health concerns and fear of unpleasant side effects are dominant reasons given by women for non‐use and for stopping a method (Sedgh and Hussain 2014). In the Nairobi data, appreciable minorities, ranging from 12 percent to 20 percent, believe that specific methods might cause serious damage to health and over half of the sample associated injectables and implants with unspecified side effects. Despite this prevalence of beliefs of undesirable health effects of specific contraceptive methods, neither of these two items showed a net effect on choice of future method in the regression modelling. Even when potential confounders (menstrual disruption, safety for long term use, and infertility fears) were omitted from the model, the effects of unspecified health concerns and side effects remained statistically insignificant. We can offer no compelling explanation for this result, but it is possible that women intending to use a method regard side effects and possible health problems as an inevitability. Moreover, that health concerns do exert an influence on method choice is confirmed by the net effects of more precisely specified method attributes, namely menstrual disruption, safety for long term use, and risk of infertility. Evidently, detection of the influence of method‐specific health concerns requires more focused and concrete measurement. In particular, the belief that a method does not interfere with regular menses had a large net effect (odds ratio slightly over 2). While this result is consistent with evidence that African women value regular menses (Glasier et al. 2003; Hindin et al. 2013), it is nevertheless paradoxical because the two main methods, injectables and implants, are known to cause menstrual disruption, particularly in the form of amenorrhea, in large numbers of users. Qualitative studies may be needed to resolve this conundrum. Views that a method is safe for long term use and does not cause infertility problems were also associated with method choice at a statistical confidence level of about 95%. Worries about permanent impairment of childbearing potential have been documented elsewhere in Africa (Hindin et al. 2013; Williamson et al. 2009; Castle 2003). Such concerns are likely to be more of a deterrent to use and selection of methods among younger women wishing to space or postpone childbearing than among those wanting to limit family size. The view that prolonged use without taking a break is unsafe is less well documented (Henry 2001). Its contribution to contraceptive discontinuation could be important and further study is justified.

Overall, among current non‐users the attributes that were associated with future method choice among women in Nairobi slums were satisfied past use, a positive experience of use by a woman's social network, husband/partner's approval, lack of interference with menses, and perception of safety for long term use. We do acknowledge that intentions are a poor substitute for behavior, though they are predictive of subsequent adoption (Curtis and Westoff 1996; Roy et al. 2003). When the follow‐up survey data become available, we will be able to assess the predictive power of method‐specific perceptions on contraceptive use dynamics, including method‐specific uptake and method‐specific discontinuation.
